# Supplementary material for: Serotonergic modulation of visual neurons in Drosophila melanogaster
Source: PLoS Genet. 2020 Aug 31;16(8):e1009003. doi: 10.1371/journal.pgen.1009003 (PMC7485980; doi:10.1371/journal.pgen.1009003)
Supplement: S1 Table — (PDF) [file pgen.1009003.s012.pdf]

S1 Table. RNA-Seq Serotonin Receptor TPMs, averages and standard deviations.

	5-HT1A	5-HT1B	5-HT2A	5-HT2B	5-HT7
T1	165	271	0.028	0.024	0.034
T1	231	305	7.53	0.000	0.000
T1	150	257	0.000	0.082	0.000
Average	182	278	2.52	0.035	0.011
STDEV	43.1	24.8	4.34	0.042	0.019
	5-HT1A	5-HT1B	5-HT2A	5-HT2B	5-HT7
L2	0.006	40.9	13.3	190	8.96
L2	38.3	22.5	1.13	45.9	34.2
L2	10.4	28.3	2.99	158	3.90
Average	16.2	30.5	5.80	131	15.7
STDEV	19.8	9.40	6.55	75.6	16.2
