# Supplementary material for: Serotonergic modulation of visual neurons in Drosophila melanogaster
Source: PLoS Genet. 2020 Aug 31;16(8):e1009003. doi: 10.1371/journal.pgen.1009003 (PMC7485980; doi:10.1371/journal.pgen.1009003)
Supplement: S2 Table — Enrichment (i.e., fold change) was calculated for cDNA from GFP-labeled cell isolates relative to pooled, unlabeled optic lobe cell isolates using the comparative CT method. (PDF) [file pgen.1009003.s013.pdf]

S2 Table. Threshhold Cycle (CT) measurements and calculated enrichments for FACS-isolated T1, L2, and L1 samples.

Target	T1 - Sample 1 (T1-split-GAL4)			T1 - Sample 2 (T1-split-GAL4)			T1 - Sample 3 (T1-split-GAL4)		
	GFP+ Ave CT	GFP- Ave CT	Enrichment	GFP+ Ave CT	GFP- Ave CT	Enrichment	GFP+ Ave CT	GFP- Ave CT	Enrichment
RP49	39.7	32.6		43.2	37.5		38.1	32.4	
5HT1A	40.3	36.1	7.29	43.3	41.7	17.1	39.4	36.1	5.64
5HT1B	41.4	36.1	3.65	44.9	41.9	6.48	41.2	36.9	2.73
5HT2A	ND	46.9		ND	ND		ND	47.0	
5HT2B	ND	38.2		ND	44.1		ND	39.5	
5HT7	ND	37.1		QC Failure			ND	37.5	

Target	T1 - Sample 4 (T1-LexA)			T1 - Sample 5 (T1-LexA)			T1 - Sample 6 (T1-LexA)		
	GFP+ Ave CT	GFP- Ave CT	Enrichment	GFP+ Ave CT	GFP- Ave CT	Enrichment	GFP+ Ave CT	GFP- Ave CT	Enrichment
RP49	37.0	32.5		37.9	31.9		38.6	33.6	
5HT1A	39.4	37.3	5.16	39.9	36.7	6.70	39.9	37.7	6.74
5HT1B	38.7	36.6	5.04	39.7	36.0	4.80	39.5	37.3	7.14
5HT2A		46.9			46.8			47.4	
5HT2B		39.0			38.3			39.7	
5HT7		37.3			36.2			37.9	

Target	L2 - Sample 1			L2 - Sample 2			L2 - Sample 3		
	GFP+ Ave CT	GFP- Ave CT	Enrichment	GFP+ Ave CT	GFP- Ave CT	Enrichment	GFP+ Ave CT	GFP- Ave CT	Enrichment
RP49	43.7	37.2		40.9	35.1		40.0	32.8	
5HT1A	ND	42.7		ND	38.2		45.9	36.0	0.152
5HT1B	ND	41.0		ND	37.7		45.7	36.6	0.262
5HT2A	49.8	49.1	57.8	ND	ND		ND	46.9	
5HT2B	48.7	43.8	3.12	45.8	42.4	5.49	45.8	38.7	1.07
5HT7	ND	42.9		44.8	40.0	2.13	ND	37.7	

Target	L2 - Sample 4			L2 - Sample 5		
	GFP+ Ave CT	GFP- Ave CT	Enrichment	GFP+ Ave CT	GFP- Ave CT	Enrichment
RP492	40.1	34.1		38.4	34.3	
5HT1A	ND	35.9		ND	40.2	
5HT1B	45.2	36.4	0.151	ND	40.1	
5HT2A	ND	ND		ND	ND	
5HT2B	44.8	37.7	0.495	ND	43.3	
5HT7	41.6	36.7	2.266	45.0	40.8	0.99

Target	L1 - Sample 1			L1 - Sample 2			L1 - Sample 3		
	GFP+ Ave CT	GFP- Ave CT	Enrichment	GFP+ Ave CT	GFP- Ave CT	Enrichment	GFP+ Ave CT	GFP- Ave CT	Enrichment
RP49	45.3	36.7		42.4	38.0		41.9	32.8	
5HT1A	ND	36.4		ND	41.5		ND	37.5	
5HT1B	ND	36.8		ND	41.9		45.7	37.1	1.416
5HT2A	ND	41.2		ND	ND		ND	ND	
5HT2B	ND	40.4		ND	43.6		ND	40.6	
5HT7	ND	37.5		ND	45.1		ND	39.1	

n
