# Supplementary material for: Serotonergic modulation of visual neurons in Drosophila melanogaster
Source: PLoS Genet. 2020 Aug 31;16(8):e1009003. doi: 10.1371/journal.pgen.1009003 (PMC7485980; doi:10.1371/journal.pgen.1009003)
Supplement: S3 Table — (PDF) [file pgen.1009003.s014.pdf]

S3 Table. Fly Strains used in this study.

Drivers	Source	Figure
5-HT1A-T2A-GAL4 MI01468	H. Dierick (Baylor)	S1B, S3E, S3D, S6B-E
5-HT1A-T2A-GAL4 MI01140	H. Dierick (Baylor)	1A
5-HT1A-T2A-GAL4 MI04464	H. Dierick (Baylor)	S3G
5-HT1B-T2A-GAL4 MI05213	H. Dierick (Baylor)	1B, S1C, S5A-F
5-HT2A-T2A-GAL4 MI0459	H. Dierick (Baylor)	S1C, S4B
5-HT2A-GAL4 MI03299	H. Dierick (Baylor)	S4A
5-HT2B-T2A-GAL4 MI5208	H. Dierick (Baylor)	1D, S1E, S3A
5-HT7-GAL4 MI00215	H. Dierick (Baylor)	1E, S1F, S3B-C, S3F, S3H
T1-spGAL4	A. Nern (Janelia)	4D, S7A, S9C-D
L2-spGAL4	L. Zipursky (UCLA)	2B, 3C-D, 4B, 5A-E, 6A, S7B, S8A, S9A
L1-spGAL4	A. Nern (Janelia)	4C, S7C, S9B
T1-LexA	A. Nern (Janelia)	2A, S7A, S8A, S8E
L2-LexA	RRID:BDSC_52510	S8B-C
L1-LexA	A. Nern (Janelia)	S8D
SerT-Gal4 (P{GMR50H05-GAL4}attP2)	RRID:BDSC_38764	S8B-E
Chat-LexA MI	RRID:BDSC_60319	S3A
GAD1-LexA	RRID:BDSC_60324	S3E

Reporters and Mutants	Source	Figure
UAS-mCD8::GFP	RRID:BDSC_5137	2A-B, 3D, S4B, S5A, S5C-F, S6B-E, S7A-C
UAS-mCD8::RFP, LexAop-mCD8::GFP (x)	RRID:BDSC_32229	S1B-F, S3A, S3E
UAS-MCFO-1	RRID:BDSC_64085	1A-E, S3B-D, S3F-H, S4A, S5C
UAS-GCaMP6f	RRID:BDSC_42747	4B-D, 5A-E, 6A-F, S9A-C
UAS-ArcLight	RRID:BDSC_51056	S9D
(sybGRASP) UAS-nSyb::GFP1-10, LexAop-CD4:GFP11	RRID:BDSC_64314	S8A-E
5-HT1A::GFP	Y. Rao (Peking U.)	3A-B
UAS-5-HT2B::GFP	Y. Rao (Peking U.)	3C
5-HT2B-GKO-GAL4	Y. Rao (Peking U.)	6B-F
