# Supplementary material for: Serotonergic modulation of visual neurons in Drosophila melanogaster
Source: PLoS Genet. 2020 Aug 31;16(8):e1009003. doi: 10.1371/journal.pgen.1009003 (PMC7485980; doi:10.1371/journal.pgen.1009003)
Supplement: S4 Table — (PDF) [file pgen.1009003.s015.pdf]

S4 Table. Primer sequences and mRNA target information.

Primer ID	Gene Symbol	Exon Junction	Product Length (bp)	Primer Sequence (5' to 3')	Primer Length	One Primer Spans	Transcript Specificit	Design
5HT1A Forward	Dmel\5-HT1A	2027/2028	159	TTCGTGGCCTGCCTAGTAAT	20	Yes	All	BLAST
5HT1A Reverse	Dmel\5-HT1A	2027/2028	159	CCAGTAACGATCGACGGCAA	20	Yes	All	BLAST
5HT2B Forward	Dmel\5-HT2B	302/303	135	AAAGCCGATTGCTTCTCCAAC	21	Yes	All	BLAST
5HT2B Reverse	Dmel\5-HT2B	302/303	135	GATTCAGGACTCGCGAAAGG	20	Yes	All	BLAST
5HT1B Forward	Dmel\5-HT1B	2148/2149	129	ATTTCGCCAGTTTGGCCATT	20	Yes	All	BLAST
5HT1B Reverse	Dmel\5-HT1B	2148/2149	129	CGTTGCTGGTGCGATAATCA	20	Yes	All	BLAST
5HT7 Forward	Dmel\5-HT7	700/701	117	TCGACGACTTTTGGAAGCAC	20	Yes	All	BLAST
5HT7 Reverse	Dmel\5-HT7	700/701	117	ATTGTCGTCGGGAACTGGG	19	Yes	All	BLAST
5HT2A Forward	Dmel\5-HT2A	480/481	186	CGACTGCAAAATGTGGGTCT	20	No	All except F	BLAST
5HT2A Reverse	Dmel\5-HT2A	480/481	186	CCTCGCAGATGTGTCTGTTG	20	No	All except F	BLAST
RP49 Forward	Dmel\RpL32	28/29	147	GGTTTCCGGCAAGCTTCAA	19	Yes	B Only	BLAST
RP49 Reverse	Dmel\RpL32	28/29	147	TGTTGTCGATACCCTTGGGC	20	Yes	B Ony	BLAST
