# Supplementary material for: Serotonergic modulation of visual neurons in Drosophila melanogaster
Source: PLoS Genet. 2020 Aug 31;16(8):e1009003. doi: 10.1371/journal.pgen.1009003 (PMC7485980; doi:10.1371/journal.pgen.1009003)
Supplement: Attachment — Submitted filename: PLOSGEN RESPONSE v54 renum PlsClnNum.docx [file pgen.1009003.s016.docx]

**Respons­­e to Reviews | PGENETICS-D-20-00681**

We thank the Reviewers for their very thoughtful feedback and for their help in improving the manuscript. Their comments are shown below in italics, followed by our point-by-point responses in plain text. As requested on the PLoS Genetics website, we have included a “clean” version of the manuscript as well as a marked-up version. Revisions are indicated below by line number and also shown in the marked-up version of the manuscript using “Track Changes”. Since the line numbers in the original and revised manuscripts are different, we refer to the line numbers in the revised, marked-up version as “Mark-Up Line #”. Unfortunately, when scrolling through the document in Track Changes, these numbers shift. We therefore designate most line numbers as approximate ranges or “~”. We also provide the analogous line numbers for the text in the “clean” copy of the manuscript (“Clean Copy Line #”).

*Reviewer #1: In this paper, Sampson and colleagues investigate how serotonin affects early processing in Drosophila’s visual system. They show that several early processing neurons express different serotonin receptors, which are localized to medulla neurites. They then use bath application of serotonin and serotonin receptor mutations to investigate the functional effects of serotonin on these early visual neurons. The experiments were careful and interpreted in a measured way. Still, there were a few aspects of the research that should be addressed.*

*Major comments:
These concerns relate to the functional characterization of L2 calcium responses in the presence of serotonin.
1) The presence of TTX in serotonin washes, as the authors also note, is unlikely to isolate cells from the network, since many or most cells in the early visual system don’t spike. Instead, a cocktail of fast neurotransmitter receptor antagonists (against GABA receptors, gluCl, and nicotinic and metabotropic Ach receptors) seems like it would largely do the trick, and eliminate some of the hedging that the current experiments require about cell autonomy of the increased-calcium effect, which appears several times in the manuscript (for instance, Line 486).*

Application of an inhibitor cocktail rather than TTX is an excellent suggestion and we will use this approach in future experiments. However, in the interest of returning a revision in a timely manner, we have not repeated the experiments in Fig 4 using this approach. As in the previous version of the manuscript, we qualify our conclusions about the cellular site(s) of serotonin’s effects. However, as both reviewer 1 and 2 noted, the focus on serotonin’s cell autonomous effects was previously overemphasized since our data does not adequately address these issues. We have reduced this emphasis in the resubmission.

*2) F6A and F4B show rather different responses to the serotonin and saline, although the genotypes are the same: in F6A the +serotonin trace does not go as high and the saline trace increases, compared to the F4B traces. The increase in the saline condition here but not earlier is especially odd and cannot be explained by the difference in TTX. What explains this variability and how does it affect interpretation of the results?*

To address this question, we first reanalyzed the data to confirm that the difference was not an artefact of our analysis. Reanalysis using updated parameters (normalizing for the earliest timepoints) is shown in the revised figure still shows a difference between 4B and 6A. We speculate that visual input may be responsible for the observed difference*.* In Fig 4B, the flies experienced no changes in their visual environment during the experiment: they were continuously shown the same, medium luminance screen. By contrast, the flies in 6A were exposed to high contrast, dark and light flashes throughout the experiment. We suggest that the increase in intracellular calcium seen in 6A saline-perfused animals could represent an adaptive response to repeated, relatively intense stimuli. Although this remains speculative, we have added text to the revised manuscript to highlight the differences between 4B and 6A and the possibility that visual stimuli may drive changes in basal calcium (Mark-Up Lines ~2700-3000; Clean Copy Lines 692-699).

*3) Fig 5D: biggest effect appears to be in rebound amplitude (i.e., the second lobe). Is that statistically significant?*

We have now analyzed the amplitude of the second calcium event/lobe for both the Fig 5 and Fig 6 data sets. We did not detect significant differences, either between saline vs. serotonin perfusion or between the 5-HT2B+/- vs. 5-HT2B -/- flies. The analysis is now included as panels A and C in both S10 and S11. We note that the amplitude of the initial decrease or increase in calcium represent previously characterized responses to a light or dark flash. The amplitude of the second event/lobe likely reflects a more complex integration of the cell’s response to both the transient “ON” and “OFF” steps. It is possible that integration of these opposing responses could obscure potential differences in the second event/lobe of the response.

*Also, it would be important to note in the paper that the indicator itself is a nonlinear function of Ca, so changes you do see can be difficult to attribute to changes in cell activity, rather than changes in Ca-indicator regime. For instance, raising baseline Ca likely changes the gain of this indicator.*

We have added this important caveat to the text (Mark-Up Lines ~2550-2750; Clean Copy Lines 674-677).

*4) In F6D, the panels show only differences from the first minute. However, by far the largest differences here appear to exist between the genotypes in epoch 1. These large baseline differences should be explored, and it seems that baseline differences among genotypes make it far more difficult to interpret the changes that occur in the presence of serotonin. This interpretation should also be addressed.*

There are indeed major differences in the baseline responses to light and dark flashes in 5-HT2B +/- and 5-HT2B -/- flies. In hindsight we should have addressed this in the text. We now do so and acknowledge that these differences could complicate our interpretation of the data, especially if the developmental absence of 5-HT2B broadly changed the physiological response characteristics of L2. We have added following caveats:

In the Results we state: “During epoch 1, the period before serotonin perfusion, the magnitude of both the dark-induced calcium increase and light-induced decrease was larger in 5-HT2B +/- compared to 5-HT2B -/- flies (compare Fig 6C and Fig 6E). Further experiments will be needed to determine the origin of this effect and whether it could distort the cells response to serotonin.” (Mark-Up Lines ~1250-1350; Clean Copy Lines 516-520).

In the Discussion we say “Additional experiments will also be needed to assess the potential developmental effects of the 5-HT2B mutant, and in particular how loss of 5-HT2B may have altered the baseline response of L2 to visual stimuli in the absence of exogenous serotonin” (Mark-Up Lines ~2200-2400; Clean Copy Lines 601-603) and “We observed differences in the visual responses of 5-HT2B +/- and 5-HT2B -/- even before serotonin perfusion. This might result from the activation of 5-HT2B by endogenously released serotonin or developmental defects caused by loss of 5-HT2B. It is possible that such developmental defects could broadly disrupt the physiology of L2 neurons and thereby confound our interpretation of the cell’s response to exogenous serotonin. Future experiments to inducibly block the activity of 5-HT2B and/or endogenous serotonin in the adult but allow wild type activity during development will be needed to address this possibility.” (Mark-Up Lines ~2600-2900; Clean Copy Lines 681-688).

*5) Last, I’m not sure I understand the logic of why the +/- would create a “sensitized genetic background”, or even how the experiments support this claim. The comparison made in the paper is between +/- and -/- with serotonin added (Fig 6), compared to +/+ with and without serotonin (Fig 5). I would expect a sensitizing background to be +/- with and without serotonin, which then shows phenotypes when +/+ did not. The comparison of +/- (+ser) with -/- (+ser) seems to have a quite different set of interpretations for the origins of the differences. The paper should make clearer how different these interpretations are in Figures 5 and 6.*

We used the term, possibly incorrectly, to designate a homozygous loss of function mutation that can reveal effects that are not obvious in the wild type background. For example, for the detection of a phenotype caused by a second null mutation in *C. elegans* described in Fleming TC et al (<https://pubmed.ncbi.nlm.nih.gov/16207764/>). However, most of the *Drosophila* literature emphasizes the use of partial loss of function alleles or haploinsufficiency in a heterozygote to generate a sensitized background for genetic interaction experiments. Therefore, to remove the possibility of confusion, we removed the term “sensitized background” from the manuscript.

Minor comments:
*1) Not too much is known about L2 functional properties, but one salient feature is surround inhibition, which was reported to rely on presynaptic inhibition of PRs (Freifeld et al., 2013). Are there experiments to do or speculation to raise about how serotonin might or might not affect this aspect of L2 function, given its actions in other systems? For instance, does raising the baseline calcium make inhibitory conductances drive stronger currents?*

We would expect that if rising [Ca] is accompanying a tonic membrane depolarization, moving further from Cl^-^ reversal potential, then the driving force on GABA-A mediated Cl^-^ current would increase. The effect of increased GABA mediated inhibition on L2 would be expected to change the spatial structure of surround inhibition as well as tip the balance between ON and OFF response components to favor ON stimulation (Freifeld L et al, Neuron 2013, 78:1075-89.) We have added this speculation to the Discussion (Mark-Up Lines ~2900-3100; Clean Copy Lines 700-707). A future experiment could test these hypotheses by using local stimuli to assess individual receptive fields, while co-expressing GCaMP6f and Rdl-FlpStop to knock out the Rdl subunit of the GABA-A receptor (Fisher YE et al, Elife 2017; 6: e22279; Keles MF et al. Cell Rep. 2020; 30:2115-24)

*2) L5 image in Fig. 1E does not show the medulla axon stratification, which would more convincingly identify L5, if you have such a case. This particular image has a lot of other expression right where one wants to see it. There is not a really good view of axon terminals in the supp fig, either.*

We were unable to find isolated images of the L5 terminals using MCFO in any of our preparations due to overlap with other more frequently labeled cell types in the medulla neuropil. Therefore, we attempted to distinguish L5 from L4 based on the absence of co-lateral terminals for L5 in the lamina neuropil. However, in absence of additional data, we are more cautious in labeling this cell as L5 in the revised manuscript. We now state: “We therefore suggest that 5‑HT7 *may be* expressed in the one remaining lamina monopolar cell subtype, L5 cells. Additional examples of 5‑HT7>MCFO labeled cells *possibly* representing L5 are included in S3B-C Fig.” (Mark -Up lines ~380-390; Clean Copy Lines 158-161). In addition, in the legend to Fig 1, we now describe panel E as “5‑HT7>MCFO‑1 labeled neurons *possibly* representing L5 lamina monopolar cells.” (Mark-Up Line ~398; Clean Copy Line 169).

*3) Enrichment figure S7 – it seems that one might label y=1 as an important point, since that’s potentially a null hypothesis. 0 is not necessarily the null hypothesis with these experiments.*

We have added a line indicating y=1 in the figure and explain the significance of this in the legend.

*4) Line 578: The first, but not. How about, "This study is the first using the modern genetic toolkit."?*

We agree with both Reviewers 1 and 2 that our previous description was awkward. This sentence has been removed in an effort to reduce the length of the discussion as requested by both Reviewer 1 and Reviewer 2.

*5) As written, the discussion contains a lot of summary of results, rather than building on them or putting them into context. Especially paragraphs 2 and 3. Around line 660, for instance, the text mostly restates points already made in the results.*

We have rewritten the Discussion section.

*Reviewer #2: Summary
In this paper, the authors investigate serotonergic processing in the visual periphery of Drosophila. So far, octopamine has been shown to have a functional role in modulating vision, and we know that a range of other amines and peptides are involved, but their roles are unclear. Serotonin has been linked to the visual modulation of circadian circuitry, and the ability to verify such a link and identify biophysical mechanisms involved is an exciting possibility raised by this work.*

*The authors used MiMIC lines and adult multicolor flip outs to identify neurons expressing serotonin receptors in the optic lamina of Drosophila. They identified that L2 expresses 5-HT2B receptors, T1 expresses 5-HT1A,B, and L5 expresses 5-HT7 (Fig. 1). They also saw expression in other neurons corresponding to C3, Mi1, TmY3 and glia adjacent to the lamina basement membrane, proposed to play a role in modulating the extracellular space of individual lamina cartridges. They confirmed expression of these receptors in L2, and T1 using RNA-seq (Fig. 2). They used targeted GFP expression to confirm that T1 and likely L2 receptor expression is in medulla layer 2, not the lamina (Fig. 3). There was no sybGRASP signal in medulla layer 2, indicating that serotonergic modulation may be paracrine signaling. Bath application of 5-HT increased baseline calcium fluorescence over minutes in the presence of TTX to block excitation via sodium channels in all neurons (Fig 4). Bath application without TTX did not appear to substantially affect responses of L2 neurons to dark (preferred stimulus) flashes, except possibly in the duration of responses (Fig. 5C’). In 5-HT2B-/- mutants, bath application of serotonin had no effect different from saline, but 5-HT2B+/- control flies showed an larger and faster responses to ON and OFF flashes (Fig. 6).

Overall, I am excited to be reading about how serotonin may modulate peripheral visual processing in Drosophila. The paper covers a lot of ground and techniques, and the authors should be proud of having done so much. I have limited my input to my own areas of knowledge. I have concerns about how the imaging data is analyzed and can be interpreted (comments).*

*The authors tried to link serotonin signaling to rapid visual processing (100 ms stimuli) but they used extremely high bath concentrations to see a modest effect on the signal processing. Rather, the pre-existing link to others aspects of neuromodulation, e.g. circadian regulation to pick one example, would seem a more likely avenue, and one in which modest serotonin levels could alter L2 processing in less pronounced but no less important ways, fitting with their biggest effect seeming to be on baseline calcium levels over minutes.*

We have modified the Introduction and Discussion to emphasize other potential roles for serotonergic signaling including circadian regulation. We have also deemphasized the potential role of serotonin in regulating calcium transients. This was done both to respond to the Reviewer’s concerns and also as a consequence of reanalyzing the data using the more stringent parameters suggested by the Reviewer. We are sincerely thankful for this input, and opportunity to improve the quality of our analysis as well as the manuscript as a whole.

*In its writing, the manuscript lacked clarity in its aims and achievements. The introduction was vague in important places and the discussion very long for what it said. I have given a list of feedback about how the manuscript could press its advantages better in a list of Writing Comments that the authors should feel free to use entirely as they like. The manuscript reads like it has had many review comments already, and the purpose of this section of feedback is to help clarify the message not add to reviewer fatigue, I hope they are helpful.*

These suggestions were indeed very helpful and we have incorporated most of them into the text. See point-by-point responses below.

*Comments
• I had issues with how the imaging data is analyzed and presented.
o Why is it not the baseline F in Fig 5A? Clearly the baseline F is varying.*

Figs 4B-D, 5A and 6A-B all show recordings that are normalized to an initial baseline, which allows the reader to directly compare the values across figures. Fig 5A could indeed show the raw F values--the traces would look identical, but the numbers on the y-axis would change and would no longer be comparable across figures.

*I don’t understand why the baseline F can’t be calculated as the F in the few seconds before the stimulus, rather than the mean of the between stimulus activity in epoch 1 (L405).*

If we normalized each flash response to the ‘local’ baseline as usual (i.e ΔF/F = (F(t) – F0)/F0, where F0 is the mean of the intensity in a short interval preceding the stimulus), for a given response amplitude, any increase in F0 would result in a lower ΔF/F value, independent of the dynamics of the indicator. We avoided doing this because we wanted to be able to show potential serotonin-induced changes in both the basal calcium levels and the transient responses to visual stimuli.

*This issue is manifest in Fig 6A where the baseline has a substantial drift with saline. The reason that DF/F is used is because baseline F drifts. This version of DF/F, normalizing to initial F, is odd and is a halfway house between a real-time updated DF/F and baseline F.*

We recognize that our method was non-standard. The method suggested by the Reviewer makes sense and would likely suffice for recordings in which changes in the baseline were due only to drift. However, in our experiments, in which the real-time baseline signal increased over time, using the suggested method would convey a decrease in the stimulus-induced response that did not fit with what we observed in the raw traces; namely, in our recordings the transient flash responses tended to increase as the baseline increased (Fig 5A). We tried to capture this phenomenon by normalizing using a non-standard method.

*Fig 6B is referred to as ‘baseline’ (L483) but y-axis is DF/F.*

In retrospect we used the word “baseline” to represent too many different things in the manuscript. In the sentence noted by Reviewer 2, we used “baseline” to refer to **basal intracellular calcium** in the absence of visual stimulation. However, at other points in the original version of the manuscript we used “baseline” to refer to the magnitude of stimulus-induced calcium transients in epoch 1 that occurred before the addition of serotonin.

We have now clarified the use of the term “basal calcium” in the text to indicate ΔF/F readings that follow a relatively slow time course and do not appear to be in response to visual stimuli. Using this definition, we observe a change in the “basal calcium” represented as ΔF/F in response to serotonin in Fig 4B,4C, 5A, 6A and 6B. The ΔF/F readings that occur during epoch 1 and prior to the change in the perfusion solution are now indicated as “baseline” values. They encompass baseline values for both *basal calcium levels* and the *magnitude of the calcium transients*. We describe this nomenclature in the Methods section, (Mark-up Lines ~3450-3550; Clean Copy Lines 1106-1112). We have also tried to use more specific descriptive terms when possible.

*o The duration of the response in 5C’ is more normally measured as the time for the response to decrease from 90%-10%, as the initial peak and final minimum are noisy measurements where the gradient is zero.
o Likewise decay kinetics might ordinarily be measured as exponential fits to 90%-10% decay, with the exponential time scale reported. A linear fit from the peak is odd.*

We have redone our analysis to include the 10-90% range for decay time and rise time (instead of 100% max-min) and we now use an exponential fit to the decay curves within the 10-90% range. We have remade Fig 5 and 6 using this method and removed the previous panels that used the original method of analysis.

Using the response from 90%-10% and an exponential decay changed the graphs in Figs 5 and 6 and led to a loss of statistical significance for several panels in Fig 6. After reanalysis, the only metric that was significant in Fig 6 was the trough size following the light flash stimuli. Therefore, we have further softened the claim that serotonin may regulate calcium transients.

o In 5C it is the difference in peak DF/F before and after serotonin, so the y-axis is not DF/F but (peak DF1/F1 – peakDF2/F2).

We have changed the labels on these graphs to clarify that the values shown in Fig 5C indicate the change in each variable relative to epoch 1: Epoch_n_ - Epoch1. To further emphasize that Fig 5 does not represent absolute values for each variable, but rather serves for comparing changes between groups across the time course, we have replaced the y axis with a scale bar.

*• An issue for the imaging part of the paper is the linearity of GCamp6f and GCamp6f dynamics in the presence of substantial changes in intracellular calcium. If the dynamics and range are affected, then the changes in dynamics and amplitude are the result of the change in calcium levels, not the amplitude and dynamics of the short-term responses. I am really not convinced that the amplitude and dynamics are meaningfully affected by 5HT, certainly not compared to apparent shifts in the baseline fluorescence. I have to say, I don’t know how I would address this issue in my own data. In an ideal world, I might check the results using a different reporter, e.g. one of the GCamp6f variants with a displaced linear calcium range – Badura… and Sam Wang 2014 Fig 3 is helpful. To be clear, I am not suggesting you do these experiments, more that the explanation is possible.*

The use of another reporter is an excellent idea. In the absence of this additional data, we have tempered our claim that we see changes in the amplitude and dynamics of the transients (see also response to Reviewer 1 above). We have also softened our claim that serotonin influences the calcium transients in light of our re-analysis of the data using the more stringent methods suggested by the Reviewer. We now state in the Author Summary:

“Activation of these receptors increased intracellular calcium in first order interneurons L1 and L2 and *may* enhance visually induced calcium transients in L2 neurons” (Mark-Up Lines ~101-103; Clean Copy Lines 46-47). In the Discussion, we state “However, it is difficult to rule out the possibility that the non-linear dynamics of GCaMP6f could influence and perhaps account for the differences we observe between groups. Further experiments using other reporters will be important to confirm our observations” (Mark-Up Lines ~2550-2750; Clean Copy Lines 674-677).

In the absence of additional data, a detailed discussion on the dynamics of GCaMP are beyond the scope of our paper. However, we have taken the Reviewer’s comments to heart and discussed the possible effects of high baseline calcium with several colleagues. Our discussion centered on the cooperative nature of Ca binding to GCaMP. One consequence of this is a higher affinity at higher Ca concentrations, and a slower release of bound calcium. Thus, in general, higher calcium levels would be expected to be associated with slower decay rates (and longer decay times) following a peak in fluorescence. In our data, we would predict that, as basal calcium levels rose, the decay after a dark flash might be artefactually lengthened. However, we observe a shorter decay time as Ca levels rise. This certainly does not prove that changes in the basal Ca levels had no influence on the kinetics of the GCaMP transients in our studies. It does, however, suggest that if they are indeed artefactual, they follow a trend opposite to that of at least one potential artefact.

*• In vivo measurements of serotonin give 280-640 nM, as measured using cyclic voltammetry after Chrimson stimulation of serotoninergic neurons (Borue,… and Jill Venton 2009). I don’t think those measurements are meant to be too stringent a ground truth but 100 uM is greater by x500 or so. This is lower that the 1 mM used by Roger Hardie.*

It is true that perfusion of exogenous serotonin will not reflect the endogenous spatiotemporal release pattern or the endogenous concentrations. The range in the references cited by the Reviewer are appreciated. Concentrations recently used by other labs in recent publications include:

1 mM - Alekseyenko OV, Chan YB, Okaty BW, Chang Y, Dymecki SM, Kravitz EA. Serotonergic Modulation of Aggression in Drosophila Involves GABAergic and Cholinergic Opposing Pathways. Current Biology, 2019.

100 µM - Zhang X, Gaudry Q. Functional integration of a serotonergic neuron in the Drosophila antennal lobe. Elife, 2016.

25 and 50 µM - Liu C, Meng Z, Wiggin TD, Yu J, Reed ML, Guo F, et al. A Serotonin-Modulated Circuit Controls Sleep Architecture to Regulate Cognitive Function Independent of Total Sleep in Drosophila. Current Biology, 2019.

We now cite these papers in the Results, Discussion and Methods (Mark-up Lines ~ 3400-3450; Clean Copy Lines 1030-1031) where we state: “The concentration of serotonin used is comparable to recent physiological studies applying exogenous serotonin to the *Drosophila* brain.”

We are hesitant to cite measurements of bulk extracellular fluid using cyclic voltammetry because it is unclear how they may relate to microdomain concentrations within the CNS. We do not think serotonin signals synaptically, but the concentration near release sites and within the confines of tightly pack processes is likely to be much higher than the concentrations averaged across the neuropil in contact with the relatively large surface of the FSCV electrode.

*Even so, I think there are limits to what we can say about the in vivo effects of 5-HT on signal processing.*

We agree and have qualified our conclusions in the revised manuscript as noted above. In addition, we have included additional text about the possible role of serotonin in more chronic processes in the Abstract (Mark-Up and Clean Copy Line 16) Introduction (Mark-Up Lines ~215-226; Clean Copy Lines 86-97) and Discussion (Mark-Up Lines ~2300-2600; Clean Copy Lines 632-640).

*The paper cites work from Elzbieta Pyza on serotonin as a circadian modulator of L1 and L2, and this seems plausible, especially for T1 which expresses circadian genes eg tim2 (Benna et al 2020 Curr Biol). I think using artificially high hemolymph levels is helpful for interpolating the effects of lower concentrations on slower timescales. I don’t think the paper can argue that serotonin helps L2 encode variable stimuli (implied by Abstract, L16 and use of 100 ms stimuli), but it is helpful and important to identify that serotonin is altering base levels in specific neurons*.

As noted above, we have reduced our emphasis on the possible modulation of brief (100 ms) stimuli by serotonin. We have also added text to discuss the potential role of serotonin in processes that occur over a longer time scales such as the regulation of circadian rhythms.

*• I didn’t find that Fig S3 D gave me the information I needed to identify C2 (mainly I could only see the soma), nor did panel B let me see the L5 medulla expression, which is sort of in Fig 1E but a bit awkwardly on the side. I didn’t read the criteria say in methods by which you identified the cells. I’m sure you are right but occasionally mistakes slip through, like the T2 paper.*

We are more circumspect regarding identification of C2 and L5 in the revised manuscript. We were unable to visualize C2 projections in the medulla and trace them to the cell body using MCFO due to labeling of other cells. We therefore broadly label this cell as a “centrifugal cell” that could be either C2 or C3 in the revised manuscript (see also response to Reviewer 1).

Unfortunately, we were unable to find isolated images of the L5 terminals in the medulla neuropil using MCFO due to strong labeling of other cell types in the same area (e.g. Mi1). We therefore used collateral processes in the lamina neuropil to distinguish L5 from the morphologically similar L4. In the absence of data from the medulla neuropil we are more cautious in the revised manuscript, and now say in the text “We therefore suggest that 5‑HT7 ~~is~~ *may be* expressed in the one remaining lamina monopolar cell subtype, L5. Additional examples of 5‑HT7>MCFO labeled cells *possibly representing* L5 are included in S3B-C Fig.” We have also changed the figure legend to read, “5‑HT7>MCFO‑1 labeled neurons ~~most likely~~ *possibly* representing L5 lamina monopolar cells.” To further address this point, we have now removed the “L5” arrowhead and label from Fig 1 Panel E.

*• In figure S1, the expression in layer 1 of lobula and outermost layer of lobula plate is interesting. Is this meaningful? Is vertical downwards motion processing affected?*

From our data set, it is still unclear which lobula cells express serotonin receptors and where these receptors are localized. At this time, we do not have any data for serotonin modulating lobula activity, but this would be a fascinating future direction.

*• There is some confusion about TTX, as it is presented as blocking inputs from other cells (L320), which is odd. Being a Na channel blocker, it is blocking depolarization from Na channels, in L2 and other cells. The paper refers to cells ‘unaffected by TTX (L330)’. My understanding is that Drosophila only has para sodium channels, and creates variety by alternative splicing etc, and all variants are blocked by TTX, while in mice there is e.g. Na_v1.9 which is TTX-resistant. The paper discusses that ‘graded potentials’ would be more likely to mediate indirect effects of serotonin in the presence of TTX (L360) as though these might require NA channels. It’s all a bit inconsistent across the paper and odd. The expression of serotonin receptors in glia adjacent to the basement membrane means that there could be a serotinergic regulation of the extracellular ion concentrations in every cartridge, as postulated by Shaw, and investigated and explained in detail by Weckstrom and Laughlin 2010*.

We have tried to clarify these issues in the revised text. We also added references on the glial regulation of extracellular ion concentrations and thank the reviewer for highlighting this idea. We now state “it is possible that activation of the serotonin receptors in lamina glia could indirectly influence L1 perhaps via regulation of extracellular ion concentrations” (Mark-Up Lines ~2300-2400; Clean Copy Lines 622-624).

As noted by the reviewer, there is no evidence for TTX insensitive sodium channels in the fly. With additional consultation from colleagues, we believe that most if not all indirect serotonergic inputs to lamina neurons would be blocked by TTX via: 1) inhibition of para variants required for action potentials, 2) the overall hyperpolarization of the brain caused by blockage of all sodium channels, 3) the presumptive blockage of many graded potentials. We have rewritten the text to indicate that action all action-potential driven inputs would be blocked by TTX and that graded potential influenced by para would be inhibited as well. However, we have left open the possibility that some graded inputs to L1 could be active.

*Typos
• L30. Without a functional -> without functional*

Done

*• L30. ‘severe’ reads badly – melodramatic? – but also the differences are in response to the application of unphysiological concentrations of 5-HT.*

This sentence was changed in response to the new analysis: “Flies without functional 5-HT2B, failed to show an increase in basal calcium in response to serotonin.”

*• L34. ‘…required for initiating visual processing’? Is that not photoreceptors? Clearly photoreceptors process visual signals, e.g. through adaptation.*

Changed to “involved in early visual processing”

*• L36. …sensory networks -> a sensory network*

This line was deleted in the revised manuscript

*• L57. Visual processing begins in the retina not the lamina. There is, for instance, the pupil reflex controlling photon capture in the retina, which is even before the photoreceptor.*

Changed to “early visual processing occurs in the lamina”

*• L58. They are first order interneurons, the photoreceptors are the first order neurons.*

Changed to “first-order interneurons”

*• L61. Identical -> indistinguishable.*

Done

*• L69. Also in lobula plate.*

“lobula plate” added

*• L72. Odd that you don’t cite a Nassel paper with Drosophila expression, or mention LBO5HT, which is quite well known. Agata Kołodziejczyk’s 2011 thesis done in his lab did 5HT immunostaining in Drosophila (I think), but I don’t remember seeing it published.*

We thank the Reviewer for pointing out this oversight. We now include text on LBO5HT and reference “Nässel DR. Serotonin and serotonin-immunoreactive neurons in the nervous system of insects. Progress in neurobiology. 1988;30(1):1-85. We also cite two other relevant references in the Discussion

Kolodziejczyk A, Sun X, Meinertzhagen IA, Nassel DR. Glutamate, GABA and acetylcholine signaling components in the lamina of the Drosophila visual system. PloS one. 2008;3(5):e2110.

Nässel DR, Elekes K. Ultrastructural demonstration of serotonin-immunoreactivity in the nervous system of an insect (Calliphora erythrocephala). Neurosci Lett. 1984;48(2):203-10.

*• L95. ‘…crucial for the initiation of visual information processing.’ Initiation? No. Perhaps crucial for early visual processing, but it’s still also so vague – L1 and L2 have well-characterized high bandpass spatiotemporal filters of luminance that reduce temporal and spatial redundancy and enhance contrast detection.*

We have changed “the initiation of” to “early.” We have left out the specifics of L2’s function since we have tried to deemphasize fast visual processing in the revision.

• L126. ‘subtype of cells with a soma’. Hmm. Perhaps ‘subtype of cell’ -> cell type, and ‘with soma’.

We changed “observed a subtype of cells” to “frequently observed a cell.”

*• L314. L2split -> L2 split*

Corrected

*• L322. ‘Cell-non autonomous’. The paper uses ‘cell non-autonomous’, ‘cell-non autonomous’, and even ‘cell-non-autonomous’.*

In response to another comment below we have taken out the repeated use of cell-nonautonomous.

*• L587. The lamina contain a small number of columnar (cartridge neurons) but there is quite a range of cells from accessory medulla etc innervating the lamina. In this paragraph you are repeating material from the introduction and early results and you’d be better discussing your results.*

We have removed that sentence and other redundancies in the Discussion.

*• L1105. Capitalization of journal titles.*

All of the journal titles are capitalized in the endnote library but applying either the PloS or Vancouver reference style did not fix this capitalization error. If the revised manuscript is accepted we will correct error this line by line.

*• L1118. Osford*

Corrected. We again thank the Reviewer picking up this and other errors.

*Writing comments – these are aimed at lifting the text.
• L15. ‘…highly variable stimulus space.’ You don’t explore how variable stimuli are encoded by L2, so this is an unkind opening sentence for your paper. They have other functions, including circadian modulation, maintaining circuit homeostasis and neuroprotection. These other roles are perhaps more likely landing ground for your paper because they require slow changes in neural activity, such as long lasting changes in baseline calcium.*

We agree. This sentence was changed to: “Sensory systems rely on neuromodulators, such as serotonin, to provide flexibility for information processing as stimuli vary, such as light intensity throughout the day**.”**

*• L19. ‘Mechanisms by which serotonin regulates visual neurons remain unclear.’ We knew before the paper that visual neurons expressed serotonin receptors and so they likely affected the neurons. The paper shows changes in baseline calcium with very high levels of applied serotonin, and at best minor changes in dynamics. I don’t think that making the mechanisms clearer is the question addressed by this paper. Rather, the paper explicitly addresses hypotheses addressed by other, wide-ranging data-driven studies, and provides evidence that an amine other than octopamine can modulate the physiology of peripheral visual interneurons.*

We have deleted “The role for serotonergic signaling in the *Drosophila* optic lobe and the manner in which serotonin receptors regulate visual neuron physiology remains unclear.” Instead, we now say “It remains unclear whether serotonin modulates the physiology of interneurons in the optic ganglia”.

*• L19. You use present tense to say what you have done. It makes it unclear which statements are statements of fact, and which are results you have found. The present tense makes it engaging, but you lose clarity in stating achievements.*

We corrected the tense throughout the manuscript.

*• L23. ‘…initial steps of visual processing’. Clearly the first visual interneurons are required for early visual processing. This is true for T1 and we have little idea of what it does. Rather, L1 and L2 are involved in detecting spatiotemporal luminance contrast. The photon count hitting photoreceptors covers ten orders of magnitude, but thanks to photoreceptors adaptation, pigment screening in the eye, and processing in the lamina, this is reduced to a voltage range of a few tens of millivolts, which is a stunning compression job.*

We have changed “initial steps of visual processing” to “spatiotemporal luminance contrast.”

*• L28. Be explicit – decreases the duration of responses is your result, I understood. ‘Alters kinetics’ is very vague and not helpful.*

We have changed “alters the kinetics” to “decreased the duration.” We have also tried to reduce the use of other relatively vague terms throughout the manuscript.

*• L33. The phrase non cell-autonomous is used 15 times in the paper and it’s very odd when talking about the cell’s physiological performance embedded in a network of cells. The lamina cells are all highly connected and operating as cartridge ensembles, so the idea that they might be operating cell-autonomously or non-autonomously feels like misplaced emphasis, perhaps inherited from previous work in cell biology. In this sentences you could say ‘…via other cells’ and there wouldn’t be any change in meaning (to this reader).*

We changed the text to read “interactions with other cells” and dropped the use of both cell-autonomous and cell-nonautonomous in the revised manuscript.

*• L33. The second after the Hardie paper you cite?*

We have removed “first” and “second” and now end the abstract with “These data demonstrate that serotonin modulates the physiology of interneurons involved in early visual processing in *Drosophila*.”

• L33. What does ‘functional’ mean? You use it a lot. I think you mean physiological. You don’t in any way identify a function for serotonin, such as improve signal contrast, reduce redundancy etc.

Physiological is indeed more accurate. We have changed functional to physiological throughout the revised manuscript.

• L35. The ending sentence is very vague. I think if you had written less vague opening sentences you would be able to land the abstract better at the end. My suggestion is to open with roles for serotonin in circadian modulation and maintaining circuit homeostasis, and end with saying that now the physiological basis of these roles can be studied in detail.

We have changed the opening of the abstract to better encompass other roles serotonin might play in the optic lobe and say: “Sensory systems rely on neuromodulators, such as serotonin, to provide flexibility for information processing as stimuli vary, such as light intensity throughout the day.”

We have made the last sentence in the abstract less vague by stating:

“These data demonstrate that serotonin modulates the physiology of interneurons involved in early visual processing in *Drosophila.”*

*• L39. ‘…not known.’ But linked to circadian function, as you cite. If you are going to identify a knowledge gap, you want to set out why it would be interesting to fill the gap.*

We now introduce a more specific knowledge gap in the abstract: " It remains unclear whether serotonin modulates the physiology of interneurons in the optic ganglia.” (Mark-Up Line and Clean Copy Line 18). In addition, in the Introduction we now say: “…the potential contributions of specific subtypes of serotonin receptors to circadian processes or more acute changes in the visual environment remains unclear” (Mark-Up Lines ~224-226; Clean Copy lines 95-97) and “Although two recent studies have reported serotonin receptor expression in visual neurons, to our knowledge there is no information on the physiological effect(s) of any serotonin receptor in any of the interneurons within the insect visual system” (Mark-Up Lines ~230-300; Clean Copy Lines 106-109).

*• L42. ‘…can alter…’ too vague again, be explicit.*

We now specify “increased intracellular calcium in first order interneurons L1 and L2 and may enhance visually induced calcium transients in L2 neurons.”

*• L42. See earlier comments on being ‘first’ and ‘functional’.*

Changed to “These data support a role for the serotonergic neuromodulation of interneurons in the *Drosophila* visual system” (Mark-Up Line 103-104; Clean Copy Lines 47-48).

*• L47-50. Your examples focus on signal processing, but the main focus of the retina papers you cite is on eye development and neuroprotection against excitotoxicity. Why focus on signal processing when you don’t analyze anything beyond ON and OFF flashes, and the biggest effect is in changes in baseline calcium?*

We have now expanded the Introduction to include additional text on circadian processes in the lamina and, overall, tried to decrease the previous emphasis on neuromodulation of rapid signaling.

*• L52. ‘…can modulate the response…’ be explicit about the changes you report.*

Changed to “some retinal ganglion cells express 5-HT2C and loss of this receptor enhanced the response to contrast-reversing gratings.”

*• L55. What does powerful mean? It’s an intensifier without qualification, be explicit. If it means there are lots of tools, then say that.*

We now say “*Drosophila melanogaster* provides a genetically tractable model to study visual circuit activity and regulation”

*• L85. Vague! Of course receptors can do different things. Give examples, make it grounded.*

We now include specific examples (Mark-Up Lines ~225-300; Clean Copy Lines 98-104).

*• L87. ‘…it is likely that…’ Of course activity in one cell can propagate to another. I think you are trying to set the reader up for the fact that L1 is affected by adding 5HT later, but this does not help.*

This section has been deleted.

*• L91. ‘…affects visual processing…’ Vague!*

This section has been rewritten.

*• L93. ‘functional effect’? See earlier comments.*

Changed to “physiological.”

*• L113. ‘prominent role’? If they don’t have the lamina they are blind. Is this what ‘prominent role’ means? The lamina compresses ten orders of magnitude of photon counts into 10s of mV, and uses the superposition principle to enhance luminance sensitivity, as well as a myriad of neural and non-neural mechanisms. Be explicit.*

We have rephrased this section to more clearly emphasize the choice of the lamina based on experimental tractability.

*• L116. ‘…previously characterized.’ But T1 hasn’t as you have pointed out.*

We have deleted this sentence.

*Rather, if serotonin is important for circadian input, it means you should look in the lamina, a brain region well innervated by serotinergic processes from e.g. the accessory medulla*.

We have added the phrase “and the proposed relationship between serotonin and circadian changes in a subset of lamina neurons in larger flies” (Mark-Up Line 315-340; Clean Copy Lines 130-131).

*• L120. ‘Importantly,…’ Repetition from L112. In any case, the paper is very tentative in its classification of cell types, rather than confidently stating e.g. L5.*

This redundant sentence has been removed. However, we have kept the tentative tone for identification of some cells including L5 and C2 based other comments by the Reviewers.

*• L123. ‘Although MCFO…’ belongs in methods. It reads like a riposte to a reviewer.*

This sentence was moved to the Methods section.

*• L125. ‘Using…’ Formulation of this sentence is repetitive of L119-120.*

It has been rephrased.

*• L141. I really didn’t like that every result is immediately compared to Ref2 61 and 62. It makes for a very slow reading experience. Can you not flag refs 61 and 62 at the start and say that you will discuss the comparison at the end of the section? The paper deserves to get a clear run at stating its own results.*

We have removed most of the comparisons with the previous sequencing studies in this section of the manuscript.

*• L161. ‘As noted above’. I looked and couldn’t find it. Recommend you delete this sentence.*

We have replaced this sentence with: “We detected additional, less frequent events for other columnar cells in the lamina” (Mark-Up Line ~403; Clean Copy Lines 176-177). The relatively high frequency labeling of T1, L2 and the putative L5 cells are described on Mark-Up lines ~346-347, ~357 and ~383 (Clean Copy Lines 138-139, 149-150, 154-155).

*• L191-201. This is all discussion and not results. Maybe a couple of sentences is fine, but this long stretch diluted from the impact of the results.*

This information was removed from the text for brevity.

*• L203. ‘Before embarking…’ Recommend delete this opening phrase. ‘We sought to confirm…’*

This phrase was removed.

*• 213. The conventional shortening is ‘std’, no? stdev is odd.*

Changed to std.

*• L312. It’s kind to say what the G_q/11 is, a G protein alpha subunit.*

“G_q/11_” was changed to “G_q/11_ protein alpha subunit”

*• L319. You should say that TTX is a Na channel blocker here, not later.*

This information was added as suggested.

*• L397. It’s a little bit odd to use ref 31 as the reference for L1 and L2 calcium dynamics, as though that is the first or best paper to describe the properties of L1 and L2.*

We added additional references here including:

REF 30: Clark DA, Bursztyn L, Horowitz MA, Schnitzer MJ, Clandinin TR. Defining the computational structure of the motion detector in Drosophila. Neuron. 2011;70(6):1165-77.

REF 108: Reiff DF, Plett J, Mank M, Griesbeck O, Borst A. Visualizing retinotopic half-wave rectified input to the motion detection circuitry of Drosophila. Nature neuroscience. 2010;13(8):973-8

REF 107: LAUGHLIN SB, OSORIO D. Mechanisms for Neural Signal Enhancement in the Blowfly Compound Eye. Journal of Experimental Biology. 1989;144(1):113-46.

*• L417. ‘rebound’ is a bit of a loaded way to describe it. It is an OFF response to the end of the ON stimulus. Rebound is usually used for mechanisms that are activated by hyperpolarization, and it is not clear that this is the case in the L2 calcium response.*

Another great point. We have removed the word rebound and instead call it a “calcium increase.”

*• L432. Figure legend is far too long. The point of the legend is just to enable the reader to read the figures, not explain them, give methods etc – that’s the job of the main paper.*

We have decreased the length of the figure legend as suggested but it still relatively long. In general, we agree and used shorter versions of the legend in previous iterations of the manuscript. Unfortunately, some readers seemed to be confused about the methods used and how the raw data were visualized, even though all of the details were included in the Methods and/or Results. We have therefore opted to err on the side of a more lengthy and redundant legend to reduce the possibility that future readers will be confused.

*• L477. Can you ‘blunt’ a ‘gradual increase’? Maybe ‘diminish’?*

**“**Blunt” has been changed to “reduce” in the text.

• L523. Figure legend is far too long again, very repetitive with Figure 5. You can say that the same conventions are used, where appropriate.

This figure legend text was reduced.

*• L569. 7 pages is a huge Discussion for this paper. Maybe 5 max?*

The previous discussion has been substantially reduced by removing the text that simply summarized the results. However, addressing the Reviewers’ comments increased it again to ~7. We can trim it further if needed.

*• L572. Functional -> physiological, and same comment throughout Discussion.*

We agree and “potential function” has been changed to “physiological effects.”

*• L578. ‘…to determine their potential function.’ You didn’t determine the function, so it makes the paper look weak to say you did. I think I have tried to give suggestions about what you have done above.*

See above.

*• L579. Saying you are the first after the first is very tortured – you are the second study. Nothing shameful about that and you can be proud.*

This now reads: “The only prior report of acute physiological response to serotonin in the *Drosophila* visual system described the modulation of potassium channels in photoreceptors (44)” (Mark-Up Lines ~2300-2600; Clean Copy Lines 625-627).

*• L616. Excellent! You handle this very well. You come out saying you did not find L3 expression, and since L3 expression was so strong in Davis et al, it’s very useful. Their L3 split has some expression of Lawf2, and that may explain the shared expression of 5-HT1A/B in Lawf2 and L3.*

Thank you***.***

*• L686. The ultrastructure is knowable now, in that it is available to trace in the FAFB data set.*

This is a great point. We now cite the FAFB data set and recent advancements in neurotransmitter mapping onto this data set (Mark-Up Lines ~2500-2700; Clean Coy Lines 663-666).

Zheng, Z., et al. (2018). "A Complete Electron Microscopy Volume of the Brain of Adult Drosophila melanogaster." Cell 174(3): 730-743 e722.

Eckstein, N., et al. (2020). "Neurotransmitter Classification from Electron Microscopy Images at Synaptic Sites in Drosophila." bioRxiv: 2020.2006.2012.148775.

*• L700-705. Repetition! Why am I reading this introduction to L1 and L2 here?*

This redundant text was removed.

*• L748. The last thing I read is in present tense, saying what you have done – very odd.*

We have adjusted the tense throughout the manuscript.

*• L981. Calcium imaging was performed as previously described, but reference is to the Keles and Frye paper and Keles is not an author, so it’s odd.*

We have added following references that were recently published by the Frye lab.

Keles MF, Hardcastle BJ, Stadele C, Xiao Q, Frye MA. Inhibitory Interactions and Columnar Inputs to an Object Motion Detector in Drosophila. Cell Rep. 2020;30(7):2115-24 e5.

Stadele C, Keles MF, Mongeau JM, Frye MA. Non-canonical Receptive Field Properties and Neuromodulation of Feature-Detecting Neurons in Flies. Current biology : CB. 2020.

• The methods were very nicely written – a nice clear, explicit and direct way of writing. I would have appreciated seeing examples of ROIs.

Thank you. ROIs or a single fly are now shown in green figure 4A.

*Reviewer #3: In the current study, the authors conduct a good standard research to prodvide how serotonergic signaling processes sensory information. Using a combination of MiMICs, MCFO, FACS, RNA-seq and RT-qPCR, the authors maped the expression patterns of 5-HT receptor neurons in the visual system, and found that 5-HT2B receptors are expressed in lanima monopolar cell L2; 5-HT1A and 5-HT1B are epressed in T1 cells; and 5-HT7 is expressed in L5 monopolar cells of the lanima. In addition, the authors observed 5-HT2A cells exhibit morpholgic similarity to optic glia cells. Furthermore, 5-HT2B and 5-HT1A receptors localize to layer M2 of the medulla. Functionally, the authors provide evidence of the initial visual processing cell subtypes as well as the primary site of 5-HT regualtion by in vivo calcium imaging.*

*There are several points that need to be addressed:
1) There are multiple receptors types locating to subtypes of cells in the visual system, however, where the 5-HT signal comes from is not known from the current study.*

We have added more information about the serotonergic neurons known to project to the optic lobe. We describe neuropil-specific innervation by serotonin cell cluster LP2, located in the accessory medulla, as well as the LBO5HT serotonergic neurons with cell bodies in the ventral protocerebrum (Mark-Up ~445-460; Clean Copy Lines 201-206).

*2) A previous study (Konstantinides 2018) employed FACS-SMART-seq to probe different receptors in the visual system, so as the current study. However, the resutls have a few overlaps. The authors discussed that the differences might be due to the mehods used to isolate target cell populations, but it would be better to have at least some clarification of the expression levels in the C2 neurons of the lamina neurons to reconcile the differences, since there are 3 types of 5-HT receptors reported in the previous study and none were detected.*

Our approach was biased toward high abundance transcripts, as we now discuss in the revised text (Mark-Up Lines ~2000-2300; Clean Copy Lines 571-582). The Table below derived from Konstantinides et al. shows that 5-HT1A was 30X more abundant than the other receptors in their samples. We therefore suggest that the MiMIC-based approach was sensitive enough to detect 5-HT1A in C2, but not the less abundant 5-HT1B or 5-HT7 transcripts as we explain in the revised text.

| Konstantinides et al. (Ref 71)  Data extracted from GSE103772 | | | |
| --- | --- | --- | --- |
| **GENE** | **C2_1** | **C2_2** | **C2_3** |
| **5HT1A** | **29122** | **32370** | **39130** |
| **5HT1B** | 694 | **1230** | **1115** |
| **5HT2A** | 24 | 14 | 32 |
| **5HT2B** | 19 | 42 | 167 |
| **5HT7** | **1065** | **1090** | **891** |

*3) Typos: Line 383 "decreased in the light in"
Line 607 "in the current study, Davis et al, and Konstantinides et al." unfinished sentence.*

We thank the Reviewer for picking up these errors. They have been corrected in the revised manuscript.

***Have all data underlying the figures and results presented in the manuscript been provided?*** *Large-scale datasets should be made available via a public repository as described in the PLOS Genetics* [*data availability policy*](https://urldefense.proofpoint.com/v2/url?u=http-3A__journals.plos.org_plosgenetics_s_data-2Davailability&d=DwMGaQ&c=UXmaowRpu5bLSLEQRunJ2z-YIUZuUoa9Rw_x449Hd_Y&r=8XCbUd3r3zA5lZOr43xG3LyOSc3JwgZLZbvj3mpeEa4&m=CLYTrahMP3cz0zjAc0lpXD3Livr-ylR6d5tRYDG-Jc4&s=2TYg1jlZJwEEZmG9kbSAj8tuDae3i51SCUGt4kokpbE&e=)*, and numerical data that underlies graphs or summary statistics should be provided in spreadsheet form as supporting information.

Reviewer #1: No: I could not find a data availability statement in the methods describing where data was deposited, for instance calcium imaging data or sequence data. The authors filled out that the data was all available, so this may have been an oversight or I could have missed it.*

We have now deposited all of the live-imaging data and analysis code at https://osf.io/39j4m/ (DOI: 10.17605/OSF.IO/39J4M). The RNA-Seq data (raw and processed files) are available on the Gene Expression Omnibus (GSE154085).
